# A rare case of coexistence of meningioma and glioma in a single patient: A case report from Nepal

**DOI:** 10.1016/j.ijscr.2024.109982

**Published:** 2024-07-02

**Authors:** Alisha Yadav, Prakash Regmi, Bikash Chaudhary, Sharad Sharma, Susmin Karki, Sushil Krishna Shilapakar

**Affiliations:** a Maharajgunj Medical Campus, Institute of Medicine, Nepal; b Tribhuvan University Teaching Hospital, Institute of Medicine, Kathmandu, Nepal

**Keywords:** Meningioma, Glioma, Co-occurrence of meningioma and glioma, Case report

## Abstract

**Introduction and importance:**

Primary brain tumors are less frequent as compared to metastatic brain tumors. Meningioma and glioma are common primary brain tumors. The patho-physiologies of meningioma and glioma are disparate. The concurrence of these two lesions in same patient is extremely rare, only few such cases are documented till now.

**Case presentation:**

We present a case of an elderly male presenting with sudden weakness of left limbs. He had weakness in his left limbs (muscle power was 4/5 and 4/5 respectively). There was no weakness in his right limbs. There was decreased sensation in his left hand and below left knee, while sensations of his right limbs were intact. Bilateral plantar reflex was down going. Bulk and tone of his limbs were normal. His higher mental function and cranial nerves were normal. There was no facial deviation. Cerebellar sign, meningeal irritation, clonus was absent. The rest of systemic examination findings were regular.

CT scan and MRI of brain revealed right frontal meningioma and right parietal high-grade glioma. He underwent right temporoparietal craniotomy and excision of both tumors. Histopathological examination confirmed the diagnosis of fibrous meningioma and high-grade glial tumor. He was discharged in stable condition.

**Clinical discussion:**

This case demonstrates rare phenomenon of simultaneous occurrence of meningioma and glioma in the same patient without any known predisposing factors. The exact mechanism behind this phenomenon is unclear.

**Conclusion:**

Clinicians should be aware of the possibility of co-existence of multiple primary brain tumors with different histologies in same patient.

## Highlights

• Meningioma and glioma are common primary brain tumors with different pathophysiologies.
• Simultaneous presence of meningioma and glioma is very rare.
• Primary modality of treatment is surgical excision of both the tumors.

## 1 Introduction

Primary brain tumors are less frequent intracranial neoplasms as compared to metastatic brain tumors [1]. Meningioma and glioma are common primary brain tumors accounting for 30 % and 33 % of all brain tumors respectively [2]. The pathophysiologies of meningioma and glioma are disparate. The concurrence of these two lesions in same patient is extremely rare, only few such cases are documented till now [3]. The coexistence of multiple primary brain tumors is a rare phenomenon that can be attributed to a complex process of tumorigenesis involving irradiation and potentially the association of residual embryonic tissue undergoing neoplastic transformation [4]. Meningioma and gliotic tumors can occur at the same time or even collide. This is mainly seen in some phacomatosis like von Recklinghausen neurofibromatosis, and in other genetic syndromes such as Turcot's and Sipple's syndrome. It can also happen after cranial radiotherapy [5].

Here we present a case of a patient who had both meningioma and glioma at the same time, without any prior exposure to radiotherapy or any genetic abnormalities. This report has been reported in line with the SCARE criteria [6].

## 2 Case presentation

A 71 year male (BMI = 25 kg/m^2^) presented with chief complaint of weakness of left upper limbs and lower limbs for 22 days. He developed sudden weakness of left hand associated with numbness. He was unable to hold weight in his left hand and was unaware of things slipping off his hand. Then, he developed weakness of his left foot and was unaware of slipping off his slipper. There was no history of abnormal body movement, altered behavior and memory. He didn't give history of trauma, headache, photophobia, slurring of speech, difficulty in swallowing. He did not give history of any prior comorbidities- diabetes mellitus, hypertension and family history of any malignancies.

On admission, his general condition was fair (GCS- E4V2M5). Bilateral pupils were 2 mm in diameter, and reactive to light. He was alert, conscious and well oriented to time, place, person. His vital signs were stable and within normal limits. There was no pallor, icterus, lymphadenopathy, edema, dehydration, cyanosis or clubbing. There was weakness in his left upper and lower limbs (muscle power was 4/5 and 4/5 respectively). There was no weakness in his right upper and lower limb with muscle power 5/5. Bulk and tone of his upper and lower limbs were normal. There was decreased sensation in his left hand and below left knee. However, sensations of right upper and lower limbs were intact. Bilateral plantar reflex was down going. His higher mental function and cranial nerves were normal. There was no facial deviation. Cerebellar sign, meningeal irritation, clonus was absent. His heart sounds S1 and S2 were normal with no murmur. His breathing sounds were normal with no added sounds. The rest of the systemic examination findings were regular.

**Fig. 1:**
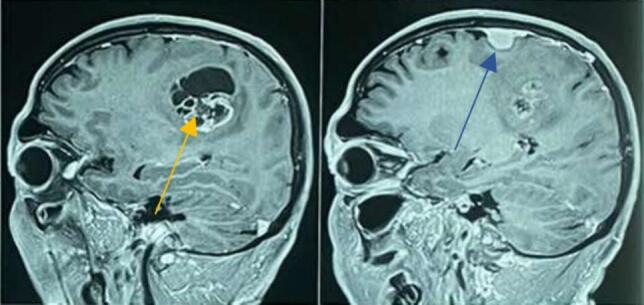
MRI brain saggital section showing Meningioma (blue arrow) and Glioma (yellow arrow). (For interpretation of the references to colour in this figure legend, the reader is referred to the web version of this article.)

**Fig. 2:**
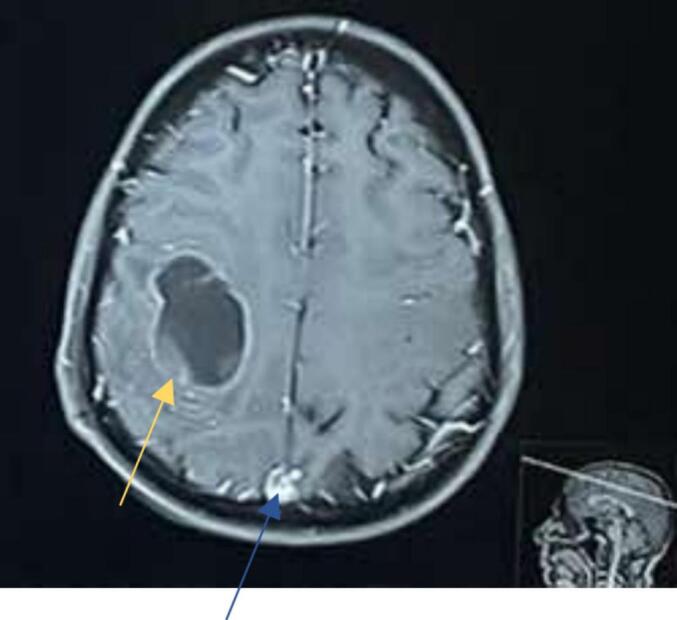
MRI brain with axial section showing Meningioma (blue arrow) and Glioma (yellow arrow). (For interpretation of the references to colour in this figure legend, the reader is referred to the web version of this article.)

**Fig. 3:**
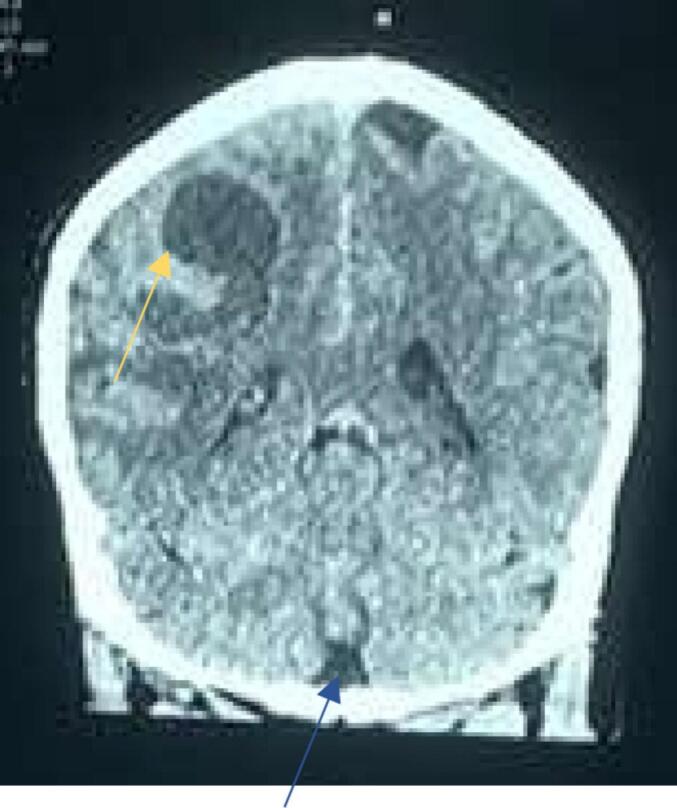
CT scan of brain showing Meningioma (blue arrow) and Glioma (yellow arrow). (For interpretation of the references to colour in this figure legend, the reader is referred to the web version of this article.)

Investigations such as hematological tests, Liver function tests (LFTs), Renal Function Tests (RFTs), Computed Tomography (CT) scan of head, Magnetic Resonance Imaging (MRI) of brain with Magnetic resonance Spectroscopy (MRS) were done as shown in Table 1. CT scan of head showed approximately 1.4 × 1.2 cm sized hyperdense extra-axial mass in right frontal cortex. A well-defined round solid cystic lesion of 4x3cm is noted in the white matter of right parietal lobe. Areas of calcification and hemorrhage were absent. Perilesional edema was seen. Mass effect was noted in the form of effacement of adjacent sulci and compression of body of right lateral ventricle. CT scan findings were suggestive of right frontal meningioma and right parietal malignant neoplastic lesion as shown in Fig. 3. MRI scan of brain with MRS revealed solid cystic mass in front-parietal region with patchy diffusion restriction, tiny foci of blooming and patchy intense enhancement of solid component with perilesional edema and mild surrounding mass effect with effacement of adjacent sulci- features suggestive of high-grade glioma as in Fig. 1, Fig. 2. T1/T2 low and FLAIR high intense and homogenously enhancing lesion in right frontal parasagittal region- likely of meningioma, as shown in Fig. 1, Fig. 2.

**Table 1:** Investigations.

Tests	Units	Results	Reference range
Sodium	mEq/L	137	135–146
Potassium	mEq/L	3.4	3.5–5.2
Urea	mmol/L	6.8	2.8–7.2
Creatinine	mmol/L	77	59–104
Glucose random	mmol/L	5.3	3.8–7.8
Total bilirubin	Umol/L	20	5–21
Direct bilirubin	Umol/L	3	<4
SGPT/ALT	U/L	22	0–50
SGOT/AST	U/L	33	0–50
Alkaline Phosphatase	U/L	72	30–120
Total protein	gm/l	65	66–83
Albumin	gm/l	43	35–52
LDH	U/L	195	0–248
TLC	/cmm	7100	4000–11,000
Hb	gm%	16.4	12–18
RBC	Million/cu	5.32	4.5–5.5
Platelets	/cumm	2,80,000	150,000–4,000,000

SGPT- Serum glutamate pyruvate transaminase (SGPT), ALT- Alanine transaminase.

SGOT- Serum glutamic-oxaloacetic transaminase, AST- Aspartate transaminase, LDH- Lactate dehydrogenase, TLC- Total leukocyte count, Hb- Hemoglobin, RBC- Red Blood Cells.

**Fig. 4:**
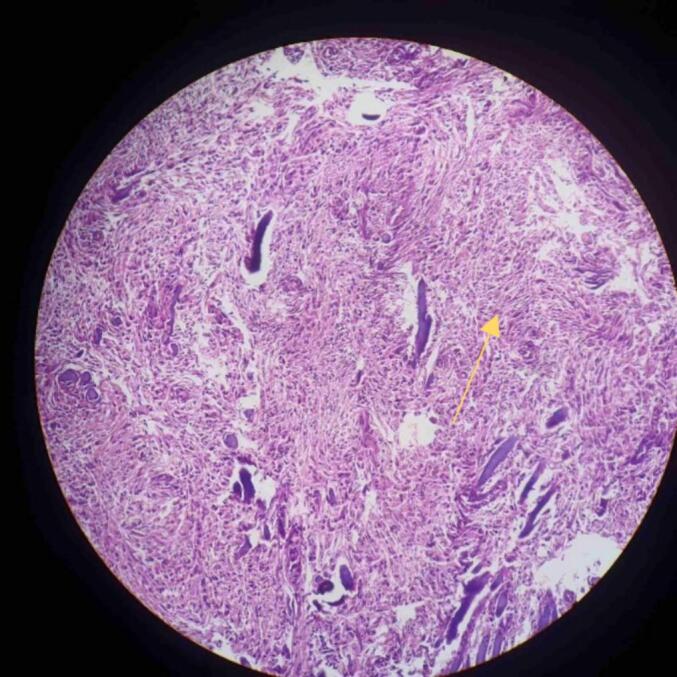
Low power view showing spindle shaped cells suggestive of Meningioma (yellow arrow). (For interpretation of the references to colour in this figure legend, the reader is referred to the web version of this article.)

**Fig. 5:**
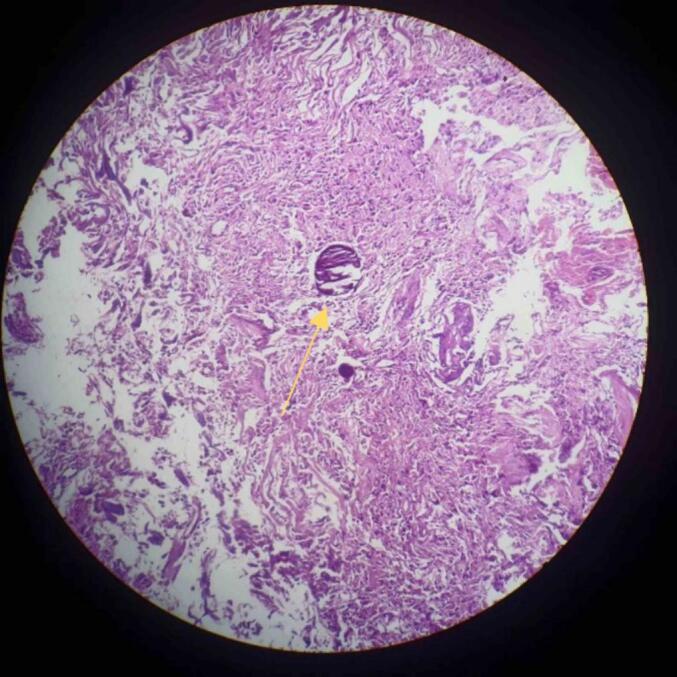
Psammoma bodies at the centre of field suggestive of meningioma (yellow arrow). (For interpretation of the references to colour in this figure legend, the reader is referred to the web version of this article.)

**Fig. 6:**
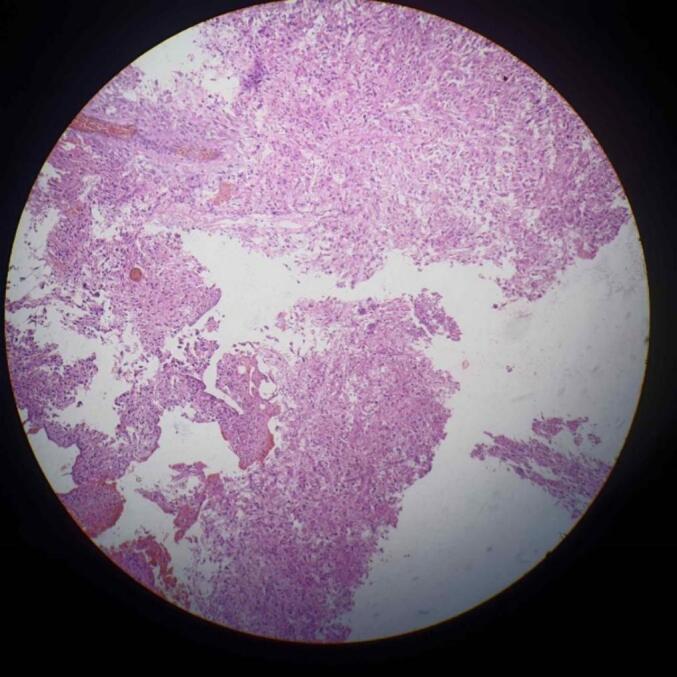
High grade glial tumor showing pleomorphism. Tumor cells are arranged in sheets. 100× magnification H n E stain.

The patient underwent right temporoparietal craniotomy. Meningioma and high-grade glioma were excised and sent for biopsy. Histopathological report confirmed it was high grade glial tumor (WHO grade 4) and fibrous meningioma (WHO grade 1) as shown in Fig. 4, Fig. 5, Fig. 6. The diagnosis of meningioma along with high-grade glioma was made. The patient was recommended to have radiation therapy, but because of the low resource setting, not all hospitals have access to radiotherapy setups, and when they do, the cost is high. As a result, the patient did not receive radiotherapy.

The patient was discharged at stable state but lost to follow up.

## 3 Discussion

The simultaneous occurrence of meningioma and glioma in the same patient with no history of radiotherapy, phacomatosis or any genetic abnormalities is very rare. The literature has reported about 67 cases of concurrent meningioma and glioma since the first case was described in 1938. The exact underlying mechanism behind this is still in controversies [7]. A patient with multiple primary brain tumors with different histology at the same time is a very rare condition. This condition can be related to radiotherapy or phacomatosis, but it can also occur without any reason [8]. In this case report, we present a case of a patient with concurrent meningioma and glioma, who had no history of radiotherapy, phacomatosis or any genetic disorders.

This rare condition has been explained by various potential hypotheses [9]. The coexistence of meningioma and glioma is more likely due to chance than to a shared mechanism that causes both types of tumors [10]. This phenomenon may be caused by some genetic factors, chemical exposure, injury, or immune system mechanism. Some believe that locally acting oncogenic paracrine factor from meningioma may affect the nearby brain tissue and glial cells and make them malignant [11]. Low-grade glioma may cause proliferative changes in the meninges. However, the exact pathophysiology of this phenomenon remains unclear [9].

The occurrence of multiple, primary brain tumors is a remarkable phenomenon. Surgical intervention is the main therapeutic option [12]. For confirmation of this rare occurrence of double primary brain tumors of different histological types, biopsy should be performed. Here, histopathological report confirmed the presence of high grade glial tumor and meningioma shown by MRI and CT scan of head.

## 4 Conclusion

Surgeons should be aware of the rare phenomenon of simultaneous presence of multiple primary brain tumors in same patient without any history of exposure to radiotherapy, phacomatosis or any genetic abnormalities. Multiple primary brain tumors can further be confirmed by biopsy.

## Author agreement statement

We the undersigned declare that this manuscript is original, has not been published before and is not currently being considered for publication elsewhere. We confirm that the manuscript has been read and approved by all named authors and that there are no other persons who satisfied the criteria for authorship but are not listed. We further confirm that the order of authors listed in the manuscript has been approved by all of us. We understand that the Corresponding Author is the sole contact for the Editorial process. He/she is responsible for communicating with the other authors about progress, submissions of revisions and final approval of proofs.

## Provenance and peer review

Not commissioned, externally peer-reviewed.

## Consent

Written informed consent was obtained from the patient for publication of this case report. A copy of the written consent is available for review by the editor-in-chief of this journal on request.

## Ethical approval

This is a case report. Therefore, it didn't require ethical approval from the ethics committee.

## Funding

The study did not receive any grant from funding agencies in the public, commercial or not-for-profit sectors.

## Author contribution

AY, BC, SS, SK: collected all the required information, reports, figures; reviewed the literature and contributed in writing and editing the manuscript. SKS and PK involved in treating the patient. All authors read and approved the final manuscript.

## Guarantor

Alisha Yadav.

## Declaration of competing interest

None.

## Data Availability

Not applicable.
